# Supplementary material for: Antimicrobial Activity of Frankincense (Boswellia sacra) Oil and Smoke against Pathogenic and Airborne Microbes
Source: Foods. 2023 Sep 15;12(18):3442. doi: 10.3390/foods12183442 (PMC10527873; doi:10.3390/foods12183442)
Supplement: Supplementary file 1 [file foods-12-03442-s001.zip › foods-2610774-supplementary/foods-2610774-supplementary.pdf]

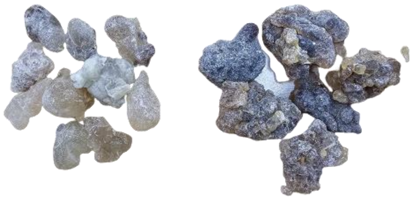

Figure S1. Frankincense oleo-gum resin; left: Hojari grdae, right: Sha'bi garde.

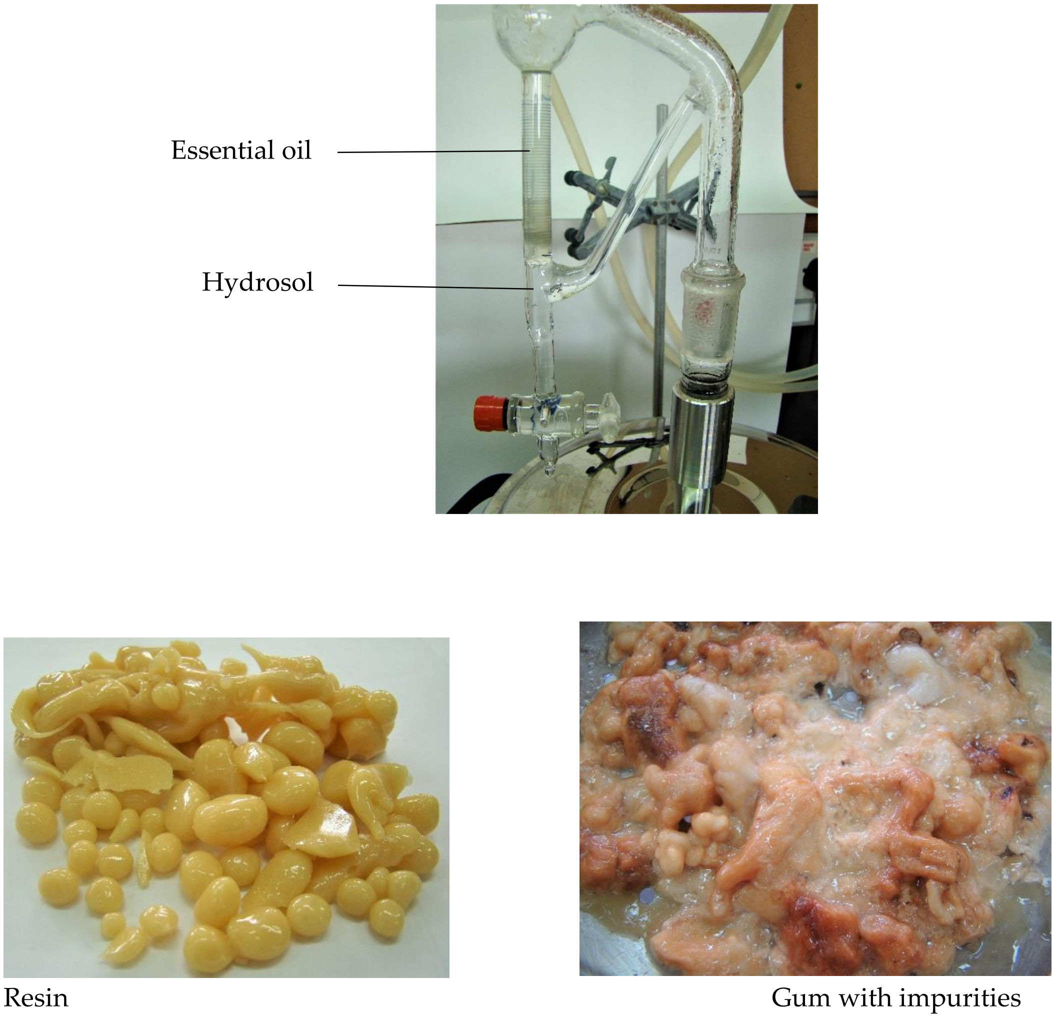

Figure S2. Frankincense (*Boswellia sacra*) oil, hydrosol (frankincense water), resin, and gum with impurities separated by steam distillation of frankincense oleo-gum resin.

Table S1. Percentages of chemical compounds detected in Sha'bi and Hojari frankincense essential oils using GC-MS and the yield of oils (% w/w).

NO	RT	Compound*	Sha'bi grade						Hojari grade				
			S1	S2	S3	S4	S5	Mean ± SD	HW1	HW2	HW3	HG	Mean ± SD
1	4.2	4-Hydroxy-4-methyl-2-pentanone	0.68	-	-	-	-	0.14 ± 0.27	-	-	-	-	-
2	6.8	α-Pinene	56.40	55.60	56.42	57.40	53.70	55.90 ± 1.23	50.70	53.80	56.97	54.65	54.04 ± 2.23
3	7.3	Camphene	9.45	9.18	9.21	9.53	9.90	9.45 ± 0.24	8.59	8.61	9.81	8.49	8.88 ± 0.54
4	8.1	β-Phellandrene	0.81	1.33	1.20	0.86	0.78	1.00 ± 0.22	1.69	0.98	1.22	1.29	1.30 ± 0.26
5	8.2	1-β-Pinene	2.47	2.41	2.41	2.41	2.46	2.43 ± 0.03	2.53	2.34	2.65	2.46	2.50 ± 0.11
6	8.7	Myrcene	0.75	0.72	0.84	0.74	0.74	0.76 ± 0.04	0.76	0.66	0.71	0.92	0.76 ± 0.10
7	9.4	(+)-3-Carene	6.69	9.82	10.21	7.76	9.07	8.71 ± 1.31	8.72	6.88	7.31	8.84	7.94 ± 0.86
8	10	*p*-Cymene	1.09	1.59	1.23	0.88	1.36	1.23 ± 0.24	1.21	1.13	1.10	0.93	1.09 ± 0.10
9	10	1-Limonene	6.55	4.79	3.78	4.37	6.13	5.12 ± 1.05	10.30	10.60	4.74	7.83	8.38 ± 2.36
10	11	*cis*-Ocimene	0.68	0.62	0.54	0.56	0.61	0.60 ± 0.05	0.40	0.97	0.60	1.01	0.75 ± 0.26
11	11	*Trans*-β-Ocimene	0.29	0.23	0.27	0.42	0.52	0.35 ± 0.11	0.31	0.45	0.37	0.47	0.40 ± 0.06
12	13	*allo*-Ocimene	-	-	-	0.29	0.33	0.12 ± 0.15	0.30	0.25	-	0.27	0.21 ± 0.12
13	15	α-campholenal	0.68	0.61	0.62	0.65	0.60	0.63 ± 0.03	0.59	0.58	0.64	0.53	0.59 ± 0.04
14	15	*allo*-Ocimene	0.37	0.36	0.33	0.33	0.35	0.35 ± 0.02	0.47	0.45	0.27	0.73	0.48 ± 0.16
15	15	*trans*-pinocarveol	1.31	1.31	1.26	1.21	1.26	1.27 ± 0.04	1.20	1.20	1.36	0.84	1.15 ± 0.19
16	16	Verbenol	1.25	1.48	1.38	1.46	1.44	1.40 ± 0.08	1.55	1.47	1.61	0.97	1.40 ± 0.25
17	16	*p*-Mentha-1,5-dien-8-ol	0.25	0.29	0.25	0.28	0.28	0.27 ± 0.02	0.22	0.25	0.26	-	0.18 ± 0.11
18	17	*p*-Mentha-1,5-dien-8-ol	0.42	0.64	0.55	0.55	0.59	0.55 ± 0.07	0.44	0.49	0.50	0.28	0.43 ± 0.09
19	18	*p*-Cymen-8-ol	0.32	0.23	0.27	0.28	0.27	0.27 ± 0.03	0.24	0.25	0.27	0.20	0.24 ± 0.03
20	18	Myrtenal	0.20	0.21	0.22	-	0.21	0.21 ± 0.01	-	0.25	0.22	0.17	0.16 ± 0.10
21	18	β-Fenchyl alcohol	0.91	0.74	0.88	0.89	1.02	0.89 ± 0.09	0.55	0.55	0.53	0.46	0.52 ± 0.04
22	18	Verbenone	0.70	0.52	0.54	0.62	0.62	0.60 ± 0.06	0.59	0.69	0.63	0.44	0.59 ± 0.09
23	19	Trans-(+)-Carveol	-	-	-	-	-	-	-	0.17	-	-	0.04 ± 0.07
24	22	Bornyl acetate	0.54	0.54	0.52	0.57	0.57	0.55 ± 0.02	0.53	0.63	0.57	0.56	0.57 ± 0.04
25	26	α-Copaene	-	0.22	0.21	0.28	0.37	0.22 ± 0.12	0.24	-	0.35	0.17	0.19 ± 0.13

26	26	β-Bourbonene	0.33	0.30	0.31	0.38	0.43	0.35 ± 0.05	0.53	0.28	0.48	0.43	0.43 ± 0.09
27	27	β-Elemene	2.44	2.67	2.62	2.93	2.21	2.57 ± 0.24	2.75	2.26	2.19	2.51	2.43 ± 0.22
28	28	*trans*-β-Caryophyllen	0.83	0.74	0.77	0.83	1.05	0.84 ± 0.11	0.88	0.91	1.06	1.09	0.99 ± 0.09
29	30	α-Humulene	0.42	0.41	0.38	0.41	0.38	0.40 ± 0.02	0.35	0.43	0.34	0.68	0.45 ± 0.14
30	31	Germacrene-D	-	-	-	-	-	-	-	-	0.34	-	0.09 ± 0.15
31	31	*Allo*-Aromadendrene	-	-	-	0.27	-	0.05 ± 0.11	-	-	-	-	-
32	31	β-Selinene	1.10	1.08	1.17	0.98	0.95	1.06 ± 0.08	1.07	0.80	1.06	0.88	0.95 ± 0.12
33	31	α-Selinene	0.54	0.55	0.64	0.58	0.52	0.57 ± 0.04	0.57	0.45	0.53	0.49	0.51 ± 0.04
34	32	δ-Cadinene	-	0.22	0.18	0.26	0.29	0.19 ± 0.10	0.21	-	0.29	0.16	0.17 ± 0.11
35	38	Hedycaryol	-	-	-	-	-	-	0.21	-	-	-	0.05 ± 0.09
36	49	Germacrene A	0.22	-	-	-	-	0.04 ± 0.09	-	0.16	-	0.18	0.09 ± 0.09
37	55	Germacrene B	1.35	0.57	0.78	1.06	0.98	0.95 ± 0.26	1.20	1.07	1.05	1.07	1.10 ± 0.06
Yield of essential oils			7.39	6.72	7.07	7.04	7.13	7.07 ± 0.24	6.83	6.92	7.40	6.58	6.93 ± 0.34

S1, S2, S3, S4, S5: Sha'bi samples 1,2,3,4 and 5, respectively. HW1, HW2, HW3: Hojari white samples 1, 2, and 3, respectively. HG: Hojari green. RT= retention time. *: compounds ordered according to retention time. -: compound not present.

Table S2. Diameter of growth inhibition zones (mm) produced by Sha’bi and Hojari frankincense oil against 12 microorganisms (values including well diameter of 6 mm).

Organism	Sha'bi grade							Hojari grade					
	S1	S2	S3	S4	S5	Mean ± SD	Rank	HW1	HW2	HW3	HG	Mean ± SD	Rank
S. aureus	20.5	15.3	13.3	15.0	13.0	15.4 ± 3.0	7	12.5	12.0	20.0	12.8	14.3 ± 3.8	6
E. coli	12.5	10.5	10.0	9.8	9.1	10.4 ± 1.3	12	11.5	10.0	10.5	9.5	10.4 ± 0.9	11
P. aeruginosa	11.8	9.5	12.3	11.8	10.5	11.2 ± 1.1	11	12.5	12.3	11.3	12.2	12.1 ± 0.6	9
Bacillus spp.	23.3	19.5	14.0	18.0	12.0	17.4 ± 4.5	5	13.0	12.8	20.0	14.3	15.0 ± 3.4	5
C. albicans	14.0	10.8	12.3	13.5	11.8	12.5 ± 1.3	10	15.8	16.5	12.3	12.5	14.3 ± 2.2	6
S. cerevisiae	47.3	36.2	40.3	36.3	29.0	37.8 ± 6.7	1	30.0	39.5	46.3	29.2	36.3 ± 8.2	1
A. flavous	13.0	9.3	13.2	15.7	13.5	12.9 ± 2.3	9	10.3	12.2	9.2	9.8	10.4 ± 1.3	11
A. ochraceus	20.2	15.3	20.5	20.2	16.8	18.6 ± 2.4	3	14.8	13.2	18.5	18.3	16.2 ± 2.6	3
A. niger	20.2	12.3	17.5	17.2	15.2	16.5 ± 2.9	6	14.7	15.5	14.0	11.2	13.8 ± 1.9	8
P. citrinum	19.0	10.7	10.7	12.7	12.3	13.1 ± 3.4	8	10.7	12.2	12.5	8.8	11.0 ± 1.7	10
A. alternata	18.8	18.2	18.3	18.7	18.0	18.4 ± 0.4	4	15.8	16.8	15.8	15.8	16.1 ± 0.5	4
F. solani	34.5	23.7	26.7	31.0	29.7	29.1 ± 4.1	2	26.2	26.3	29.0	31.2	28.2 ± 2.4	2
Mean ± SD	21.3 ± 10.3	15.9 ± 7.8	17.4 ± 8.6	18.3 ± 7.9	15.9 ± 6.8			15.6 ± 6.1	16.6 ± 8.4	18.3 ± 10.4	15.5 ± 7.4		

S1, S2, S3, S4, S5: Sha’bi samples 1,2,3,4 and 5 respectively. HW1, HW2, HW3: Hojari white samples 1, 2, and 3 respectively. HG: Hojari green. SD: standard deviation. Rank 1 represents most sensitive organism.

Table S3. Effects of Sha'bi and Hojari frankincense oil at different concentrations on optical density (OD $_{620}$) of cultures of 12 microorganisms.

Organism	Oil	Oil concentration (% v/v)									Organism
		50.00	25.00	12.50	6.25	3.13	1.56	0.78	0.39	0.20	control
S. aureus	S1	0.125	0.104	0.069	0.041	0.118	0.249	0.299	0.310	0.305	0.345
	HW3	0.108	0.066	0.069	0.047	0.156	0.249	0.287	0.291	0.295	0.382
E. coli	S1	0.472	0.139	0.048	0.045	0.047	0.037	0.249	0.402	0.463	0.492
	HW1	0.444	0.102	0.076	0.043	0.038	0.045	0.359	0.442	0.456	0.528
P. aeruginosa	S3	0.749	0.309	0.065	0.089	0.506	0.521	0.538	0.551	0.541	0.534
	HW1	0.736	0.225	0.225	0.052	0.470	0.478	0.490	0.504	0.499	0.517
Bacillus spp.	S1	0.773	0.233	0.099	0.049	0.280	0.275	0.264	0.269	0.274	0.297
	HW3	0.860	0.234	0.090	0.077	0.269	0.271	0.264	0.273	0.253	0.410
C. albicans	S1	1.249	0.343	0.105	0.045	0.044	0.791	0.903	0.951	0.928	0.963
	HW2	0.683	0.268	0.073	0.036	0.586	0.666	0.790	0.862	0.865	0.957
S. cerevisiae	S1	0.855	0.206	0.090	0.067	0.045	0.060	0.546	0.705	0.724	0.799
	HW3	1.106	0.249	0.093	0.044	0.037	0.588	0.352	0.583	0.558	0.717
A. flavous	S4	0.312	0.189	0.112	0.150	0.231	0.484	0.749	0.881	1.239	1.404
	HW2	0.515	0.219	0.183	0.168	0.196	0.286	0.350	0.649	0.640	1.033
A. ochraceus	S3	0.498	0.277	0.116	0.099	0.198	0.179	0.227	0.352	0.631	0.582
	HW3	0.637	0.245	0.121	0.172	0.164	0.220	0.328	0.434	0.342	0.431
A. niger	S1	0.492	0.206	0.109	0.070	0.109	0.093	0.157	0.684	1.257	0.933
	HW2	0.620	0.232	0.117	0.075	0.080	0.162	0.579	0.869	1.083	0.797
P. citrinum	S4	0.204	0.202	0.185	0.073	0.260	0.598	0.465	0.998	1.361	1.137
	HW3	0.379	0.176	0.224	0.309	0.455	0.616	1.057	1.014	1.371	1.232
A. alternata	S4	0.916	0.148	0.143	0.077	0.140	0.221	0.224	0.345	0.500	0.511
	HW2	0.914	0.193	0.121	0.097	0.117	0.307	0.272	0.546	0.879	0.551
F. solani	S4	0.746	0.271	0.108	0.051	0.051	0.061	0.512	0.683	0.698	0.749
	HG	0.808	0.083	0.107	0.017	0.025	0.060	0.237	0.674	0.729	0.729

S1, S2, S3, S4, S5: Sha'bi samples 1,2,3,4 and 5 respectively. HW1, HW2, HW3: Hojari white samples 1, 2, and 3 respectively. HG: Hojari green.
